# The Effect of Real Time Integrated Supportive Supervision Visits on the Performance of Health Workers in Zambia

**DOI:** 10.29245/2578-3009/2021/S2.1109

**Published:** 2021-04-15

**Authors:** Abubakar Sadiq Umar, Isah Mohammed Bello, Joseph Chukwudi Okeibunor, Pascal Mkanda, Godwin Ubong Akpan, Daudi Manyanya, Shibeshi Messeret Eshetu, Masvikeni Brine, Matapo Belem, Masumbu Penelope, Daniel Fussum

**Affiliations:** 1 WHO Regional Office for Africa (WHO AFRO); 2 WHO East & Southern Africa Support Team (WHO ESA IST)

## Abstract

The use of online Integrated Supportive Supervision (ISS) is aimed to improve the quality of services provided by front line health workers. This work is aimed to document the effects of ISS on the performance of health workers in Zambia using selected key surveillance and immunization process indicators. ISS data on WHO ODK server of all Integrated Supportive Supervisory (ISS) visits that were conducted in Zambia between 1^st^ January 2018 to 30^th^ September 2018 were analysed to determine the Percentage point difference between the first and the most recent ISS visits in order to determine whether an observed gap during first ISS visit had persisted during the most recent ISS visit. Our study demonstrated that ISS has remarkable percentage point increase between the first and the most recent ISS visits on availability of an updated monitoring chart, health workers knowledge of AFP case definition and AFP case files. However, there exist variations in the frequency of ISS visits across the provinces of the country. Future research effort should consider assessing the quality of the ISS data through periodic data validation missions.

## Introduction

Integrated Supportive Supervision (ISS) is a process which encourages the delivery of quality service for optimal outcomes through better communication, team spirit to resolve identified problems, and mentoring to motivate health workers to supervise, monitor and improve their individual and collective performance. It improves the knowledge and skills of peripheral health workers in order to ensure the delivery of quality health services through direct contact with health workers for an on-site observation of health delivery processes, reviewing of registers and other documents, immediate orientation of health workers on observed gaps in knowledge and or skills and the provision of documented feedback on issues, challenges and jointly agreed action points^1,2^. Active case search and supportive supervision are among the approaches to strengthen Acute Flaccid Paralysis surveillance and immunisation service delivery. Various studies have advanced that ISS improves surveillance and immunisation service delivery in India^3–6^ Uganda^7^, Georgia^8^ and other parts of the world^9,10^. However, it is important to note that other researchers reported that ISS led to no improvement in the knowledge and skill of health workers, demotivation, persistence of observed problems^11–13^ and ultimately poor immunisation^13^ and Malaria program outcomes^14^. These assertions must be interpreted in the context of the study design and methods of data collection, and in line with study area specific prevailing local systematic problems such as poor financial remuneration, work overload, lack of transport and incentives for outreach/mobile immunisation sessions and authoritarian supervisory approach. The Governments and Partners of African countries are increasingly receiving calls to improve the health of their citizens. In 2016 the Ministerial Conference on Immunization in Africa had affirmed that universal access to immunisation is a corner stone to reduce child mortality, morbidity and disability (ADI Declaration). Moreover, immunisation will facilitate the attaining the goals of Global Vaccine Action Plan (GVAP) and Africa to be certified polio free. This requires a robust system of supervision, monitoring and evaluation that will provide real time information for decision making to health Policy makers and program Managers. One approach is the use of innovative technologies such as ISS, Auto Visual AFP Detection and Response (AVADAR), eSurv and Geographical Information System (GIS). The use of these technologies was endorsed by Ministers of Health during the 67^th^ session of the African Regional Committee meeting in August 2017 (World Health Organisation, 2017)^15^. Furthermore, Zambia is among the countries in the African region that have been conducting Integrated Supportive Supervision (ISS) using the online tools on the Open Data Kit (ODK) platform. The total number of geocoded active case search (ACS) and supportive supervisory visits increased by 5,457% increase from 14 records in January 2018 to 764 by the end of September 2018. The system also provides real-time analysis and automated alert to Program Managers which has improved decision making process and the implementation of appropriate action. Moreover, geo-coded ISS is currently a requirement by the African Regional Certification Committee (ARCC) for regional Polio free status certification.

However, there has been no systematic documentation on the effect of ISS visits on the performance of health workers. Hence, this study aims to document the effects of ISS on the performance of health workers in Zambia using selected key surveillance and immunization process indicators. The specific objectives of this study include:

- To assess the frequency of ISS visits by provinces
- To determine the percentage point difference between the first and the most recent ISS visits using key selected surveillance and routine immunisation indicators

## Methodology

### Study design

This is a descriptive cross-sectional study.

### Study area

Zambia lies in the Southern part of Africa and share common international borders with the Democratic Republic of Congo (DRC) and Tanzania to the north, Namibia and Angola to the west, Mozambique and Malawi to the east and Republic of Angola to the west, and Zimbabwe to the south. The country has an estimated population <1 year and <15 year population of 675,509 and 7,599,474 respectively. Currently there are 2,443 health facilities across the 10 and 103 provinces and districts respectively that are on the WHO AFRO server.

### Study population

This comprises of all health facilities where ISS was conducted from the 1^st^ January 2018 to 30^th^ September 2018.

### Source of data

ISS data on WHO ODK server of all Integrated Supportive Supervisory (ISS) visits that were conducted in Zambia between 1^st^ January 2018 to 30^th^ September 2018. The ISS is an integrated electronic checklist used for supervision of routine immunisation and for the conduct of active case search for Acute Flaccid Paralysis (AFP) and other diseases ear mark for eradication or elimination depending on the country need as well as to provide. The tool is administered by Government, WHO and partner staff using smart mobile phone in the field at health facilities and surveillance priority focal reporting sites. Once the tool is completed and submitted, the data will be automatically mapped on a WHO server in AFRO with time and location stamp. Responses made by the same health facility surveillance and immunisation focal persons for both the first and the most recent ISS visit was primarily considered for analysis.

### Identifying health facilities that met the inclusion criteria

From the identified health facilities in step 3 above, the health facilities were further categorized as (a) Health facilities with only one ISS record and (b) Health facilities that have 2 or more ISS records and therefore have met inclusion criteria. .

### Data analysis

Percentage point difference was calculated between the first and the most recent ISS visits in order to determine whether an observed gap during first ISS visit had persisted during the most recent ISS visit. A bivariate analysis was conducted to determine any significant statistical association between four selected variables and ISS visits with critical level set at 5%.

### Variables used to assess the impact of ISS on the performance of health workers

The variables used to assess the changes that might have occurred between the first and the most recent ISS visits include: availability of an updated monitoring chart, knowledge on AFP case definition, availability of AFP case files, availability of RED Micro-plan and unreported (missed) AFP cases.

- Updated the monitoring chart: A monitoring chart was considered to be updated if all parameters were filled correctly as of the preceding month
- Knowledge of AFP case definition: A health worker’s knowledge on case definition for AFP was considered good if in his/her explanation it included all of the following: age (<15 years), Type of paralysis (flaccid), onset of paralysis (acute, sudden), part of the body commonly affected (limbs, arms, legs).
- Availability of AFP case files.
- Availability of health facility based RED MicroPlan: A health facility (HF) was adjudged to have RED Micro-Plan if the plan included information on catchment area target population, names of all settlements, distance to the HF, session plan by strategy for each settlement, quantity of vaccines and other immunization requirement, type and cost of transport for mobile and outreach sessions, and community link activities
- Unreported/missed AFP cases are previously unreported AFP cases found in the registers (inpatient, (in-patient, physiotherapy and or medical records) during an ISS visit

A Comparison of responses between the first and subsequent ISS visits for each of the four variables was made in order to observe whether the gap persisted or resolved as a proxy for quality of the on the job training and implementation of the action points recorded following the visit (shared feedback). Hence, the number and proportion of monitoring charts that were updated after the first ISS; the number and proportion of health workers who knew the case definition of an AFP case during the second ISS visit, the number and proportion of health facilities with AFP case file and the number and proportion of health facilities with RED Micro-Plans were determined. The denominator was the total number of health facilities with more than one ISS visits, while the numerator was the number of health facilities that have improved following subsequent visit.

## Ethical Issues

The research protocol was independently reviewed and approved by the Ethical committee in the WHO African regional office, Congo Brazzaville.

## Result

During the period under review, there were 769 geocoded records ISS visits. One hundred and sixty one health facilities have had ISS visits more than once and therefore, have met the inclusion criteria with more than half of these health facilities accounted by the Copperbelt and North western provinces. The Southern province has no live ISS record on the server (Table 1).

**Figure 1:**
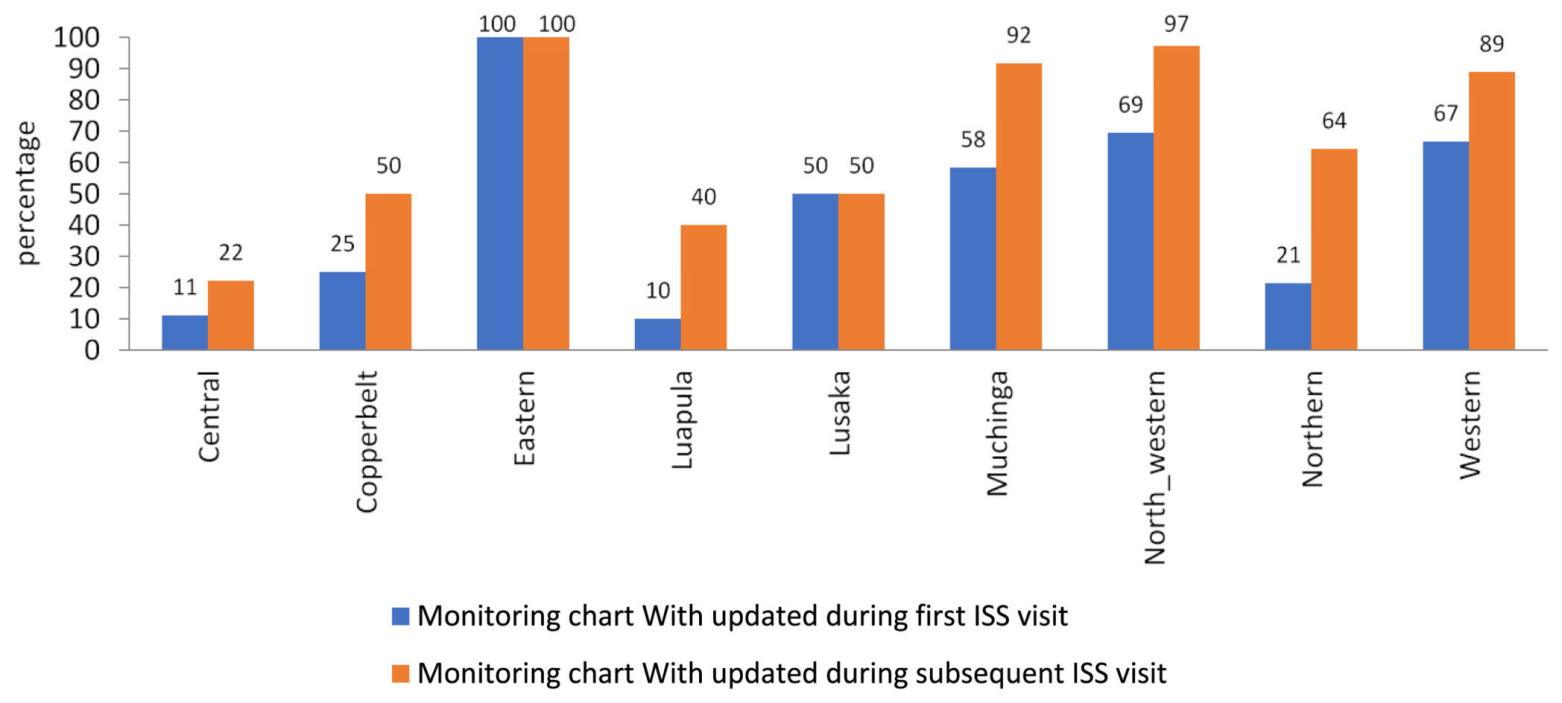
Proportion of health facilities with updated immunization monitoring chart during the first and the most recent ISS visit by province, Zambia, January - September 2018

The proportion of health facilities with an updated immunisation monitoring chart during the first ISS visit range between 10% in Luapala province to 100% in Eastern province. However, follow up ISS visits have shown that there was an increase in the proportion of health facilities with updated immunisation monitoring chart in all provinces with the exception of Eastern and Lusaka provinces. The highest increase of 53%, 34% and 30% in the proportion of health facilities with updated immunisation monitoring chart was observed in Northern, Muchinga and Luapala provinces respectively (Figure 1).

Supportive supervision aimed to improve health workers performance. There appeared to be a significant association between health facilities that have had supportive supervisory visits and having an updated Monitoring chart (χ^2^ = 29.46, df = 1; *p* <.001) (Table 2).

During both the first and most recent ISS visits, respondents in 90% of the health facilities were found to have good knowledge of AFP case definition. Respondents from the North western and Western provinces had accounted for the highest proportion of respondents (8.7%) with poor knowledge of AFP case definition (Table 3). There is a significant statistical association between supportive supervisory visits and the knowledge of AFP case definition among health worker’s in health facilities visited (χ^2^ = 33.11; df = 1; *p* <.0001) (Table 4).

**Figure 2:**
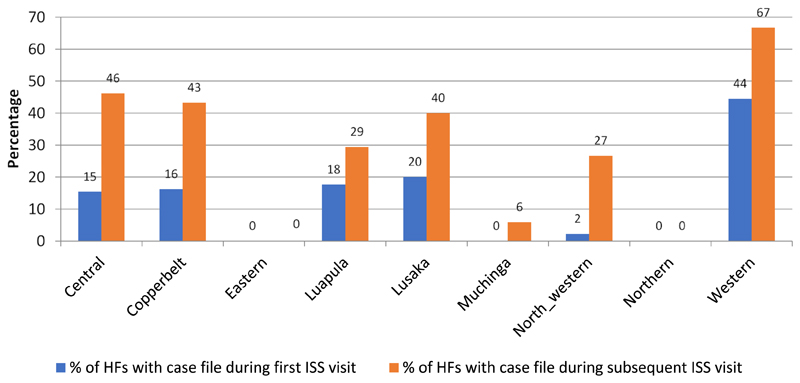
Health facilities with AFP case file during first and the most recent ISS visits by province in Zambia, January - September 2018

During the period under review, none of the health facilities in the Eastern, Northern and Muchinga provinces have AFP case file during any of the reported ISS visits. In the remaining there have been reductions in the proportion of health facilities without an AFP case file ranging from a 6 to 31 percentage point reduction in Muchinga and Central provinces respectively. The eastern and northern provinces (Figure 2).

**Figure 3:**
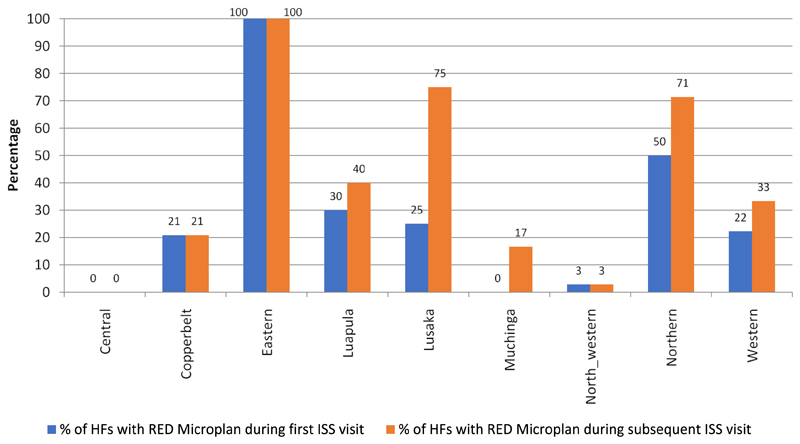
Health facilities with RED Micro Plan during the first and most recent ISS visit by province, Zambia, January - September 2018

Lusaka province had shown a 50 percentage point increase in the proportion of health facilities with a RED micro plan between first and the most recent ISS visits. Conversely, all the health facilities in the Central, Copperbelt and the North western provinces had shown no improvement in the number of health facilities with RED micro plan between the first and the most recent visits (Figure 3).

There is no significant statistical association between supportive supervisory visits conducted and the availability of RED micro-plans in health facilities visited (χ^2^ = 1.31; df = 1; *p* > .05) (Table 5).

## Discussion

In this study, we found wide variation between provinces in the frequency of ISS visits conducted despite it is a requirement for regional certification. The average of ≤1 health facility that had 2 or more ISS records (met inclusion criteria) in the past nine months in two fifths of provinces in Zambia (Eastern, Lusaka, Southern and Western) raises concern on the failure to adhere to surveillance and immunisation guideline. Several studies have similarly reported few numbers of active case search and supportive supervision were conducted in other sub-Saharan African and other developing countries with attendant risk of importation ofpolio virus^16–19^. It is important to note that our study had not explored the reasons for the few frequency of visits in provinces mentioned above due the fact that such information is not available on the ODK server; however, it might not be unrelated to lack of accountability, work overload and operational cost as was observed in similar African settings^1,12,18,19^.

Our study demonstrated that ISS has significant influence on three out of the four selected indicators namely updated monitoring chart, knowledge of AFP case definition and AFP case files. These three indicators had shown remarkable percentage point increase between the first and the most recent ISS visits. Similar positive effect of ISS on surveillance performance and routine immunisation service delivery was reported in some African countries and in other parts of the world^3–8^. The observed positive effect of ISS on the performance of health workers might be due to the basic essentials of supportive supervision such as review of records, focussed on the job training, mentoring and participatory Supervisor-Supervisee problem solving approach. Hence, considering the fact that the improvement in the three out of the four selected indicators was observed in health facilities having between 2 to 7 ISS visits, we opined that the quality rather than the frequency of ISS visits could have led to the observed positive effect on health workers performance. However, we observed that ISS visits does not have significant statistical association on the availability of RED micro-plans during both the first and most recent ISS visits. The reason could be the fact that micro-planning requires a lot of resources and involves the participation of communities to agree on day and dates of proposed outreach and mobile immunisation services, the cost of transport and allowances for the health workers. Hence, it is difficult to organise and require higher level approvals than updating monitoring charts, opening AFP case files and reading through the surveillance guidelines to understand AFP case definition. Furthermore, contrary to our findings, several studies have reported that ISS does not have positive effect on knowledge and skills of health workers^11–13,19^. The mixed effect observed in our study highlights the need for future research to explore factors such as the frequency of ISS visits, time spent per ISS visit, the knowledge and skills of the supervisors could affect the performance of health workers.

## Conclusion and Recommendations

Our findings demonstrated that ISS have positive effect on the performance of health workers. It has improved key surveillance and routine Immunization activities through systematically conduct of active case search, on the job training to address observed gaps, mentoring and positive feedback. However, in order to benefit all front line field staff not only in provinces with very few number of visits, there is the need to conduct national quarterly surveillance and routine immunisation reviews primarily driven by the Ministry of Health with a view to identify bottlenecks that had led to observed gaps and come up with an agreed action points. Furthermore, future effort should consider assessing the quality of the ISS data through periodic data validation missions.

## Public Health Implication

Our findings underscored the need to strength and scale up the use of ISS tool to provide real time information for decision making process aimed at improving and or maintaining polio certification requirements. The lack of or very few live ISS records in some provinces of the country is suggestive of weakness of the surveillance and routine immunisation service delivery to closed surveillance and immunity gaps.

## Limitations

- Our study is a descriptive cross sectional design and therefore, does not prove causality. It provided only insights on the status of some selected key functions that a health worker is expected to do, so that Supervisors and Programme Managers may tailor the required support to address any observed gaps.
- We did not explore the reasons for few number of ISS visits in some provinces which could have provided additional insights for providing support. However, it is imperative to emphasize that our findings is significant because of large sample size (769 geocoded live ISS records) spread across 103 districts in 9 out of the 10 provinces of the country.
- In the event that the health workers have access to source of information on surveillance and Immunisation, it might have influenced the observed results. However, we have analyzed only ISS records of the same surveillance and Immunisation focal points for both the first and the most recent ISS visits.

## Figures and Tables

**Table 1:** Number of health facilities that have met the inclusion criteria by province in Zambia, January - September, 2018

Province	Number of districts in the province	Total number of health facilities in the province	No Health facilities visited in the province	Health facilities with ISS data/record		
				Health facilities with only one ISS record	Health facilities that have 2 or more ISS records (met inclusion criteria)	Health facilities that have 2 or more ISS records (met inclusion criteria) - Providing RI services
Central	11	276	60	32	13	9
Copperbelt	10	346	198	41	37	24
Eastern	9	292	21	19	1	0
Luapula	11	203	87	44	17	10
Lusaka	8	208	47	36	5	4
Muchinga	7	124	74	37	17	12
North_western	9	233	128	22	45	36
Northern	9	182	89	44	17	14
Southern	13	314	0	0	0	0
Western	16	265	65	46	9	9
Grand Total	103	2443	769	321	161	118

**Table 2:** Association between ISS visits to health facilities and having an updated monitoring chart in Zambia, January - December 2018

Time of visit	Updated Monitoring Chart	
	Available	Not available
Before any ISS visit	160	149
Following ISS visit	302	158

**Table 3:** Number of health facilities whose respondent have good knowledge of AFP case definition during first and most recent ISS visit by province, Zambia, January - September 2018

Province	Number of with more than one ISS visits	Respondents knowledge of the case definition for AFP	
		Good during first ISS visit	Good during subsequent ISS visit
Central	13	13	13
Copperbelt	37	36	36
Eastern	1	1	1
Luapula	17	17	17
Lusaka	5	3	3
Muchinga	17	17	17
North_western	45	35	36
Northern	17	17	17
Western	9	4	4

**Table 4:** Association between ISS visits to health facilities and health workers’ knowledge on AFP case definition in Zambia, January - December 2018

Time of visit	Koweldge of AFP case definaition	
	Good	Not good
Before any ISS visit	98	268
Following ISS visit	190	213

**Table 5:** Association between ISS visits to health facilities and having a RED micro-plan in Zambia, January - December 2018

Time of visit	RED Microplans		
	Available	Not available	Total
Before any ISS visit	156	178	334
Following ISS visit	184	251	435
